# Case Report: Pediatric AML with *TBC1D15::RAB21* fusion and FLT3-ITD/NPM1 co-mutation: diagnostic pitfalls in morphologic mimicry of acute promyelocytic leukemia

**DOI:** 10.3389/fonc.2025.1683005

**Published:** 2026-01-13

**Authors:** Qiang Yao, Xiaoyong Chen, Meizhu Luo, Zhenhu Lin, Xiaoying Fu

**Affiliations:** 1 Department of Laboratory Medicine, Shenzhen Children’s Hospital, Affiliated to Shantou University Medical College, Shenzhen, Guangdong, China; 2 Department of Hematology, Shenzhen People's Hospital (The First Affiliated Hospital, Southern University of Science and Technology; The Second Clinical Medical College, Jinan University), Shenzhen, Guangdong, China

**Keywords:** acute myeloid leukemia (AML), APL, diagnostic pitfalls, FLT3-ITD/NPM1 co-mutation, TBC1D15::RAB21 fusion gene

## Abstract

We report a diagnostically challenging case of acute myeloid leukemia (AML) in a 2-year-9-month-old boy, presenting with diarrhea and pancytopenia. Bone marrow aspiration revealed 90% blasts exhibiting cup-like nuclei and azurophilic granules, morphologically mimicking acute promyelocytic leukemia (APL).However, immunophenotyping was inconsistent with classic APL, showing positivity for CD33 and cytoplasmic myeloperoxidase (cMPO) but negativity for CD34 and HLA-DR. Molecular analysis was negative for the canonical PML::RARA fusion but identified a rare *TBC1D15*::*RAB21* fusion, alongside FLT3-internal tandem duplication (ITD) and *NPM1* mutations. The stark contrast between the APL-like morphology and the molecular findings created a significant diagnostic pitfall, posing a risk for therapeutic misdirection. The patient achieved sustained remission following risk-adapted AML chemotherapy and allogeneic hematopoietic stem cell transplantation (allo-HSCT). This case underscores three critical points in pediatric AML: (1) the essential role of integrated molecular profiling in resolving morphologic ambiguities to prevent misclassification; (2) the complex prognostic impact of FLT3-ITD/*NPM1* co-mutations in childhood AML; and (3) the potential therapeutic efficacy of allo-HSCT for rare fusion-driven subtypes.

## 1 Introduction

Acute myeloid leukemia (AML) is a common pediatric malignancy characterized by high genetic heterogeneity, with fusion genes frequently serving as key driver events (1, 2).While specific fusion genes like *RUNX1*::*RUNX1T1* or *KMT2A* rearrangements define distinct AML subtypes and influence prognosis, a major diagnostic challenge arises from morphologic mimicry (1, 3). Blast cell morphology, particularly features like cup-like nuclear invaginations and dense azurophilic granules, is strongly associated with acute promyelocytic leukemia (APL), which is defined by rearrangements of the RARA gene, most commonly *PML*::*RARA (*4, 5). However, when these classic morphological features appear in the absence of a RARA fusion, a significant risk of misdiagnosis emerges. This refers to the potential for incorrectly classifying the AML subtype based on morphology alone, which could lead to delayed or inappropriate treatment. Mutations in FMS-like tyrosine kinase 3 with internal tandem duplications (FLT3-ITD) occur in approximately 10-30% of pediatric AML cases and are associated with high-risk disease (6–11). Conversely, concurrent *NPM1* mutations may confer a more favorable outcome (5). This report describes a pediatric AML case driven by a rare *TBC1D15*::*RAB21* fusion (12–14), which presented with striking APL-like morphology, highlighting the critical need for rapid and comprehensive genetic analysis to overcome diagnostic pitfalls and guide appropriate therapy.

## 2 Case presentation

**Figure 1:**
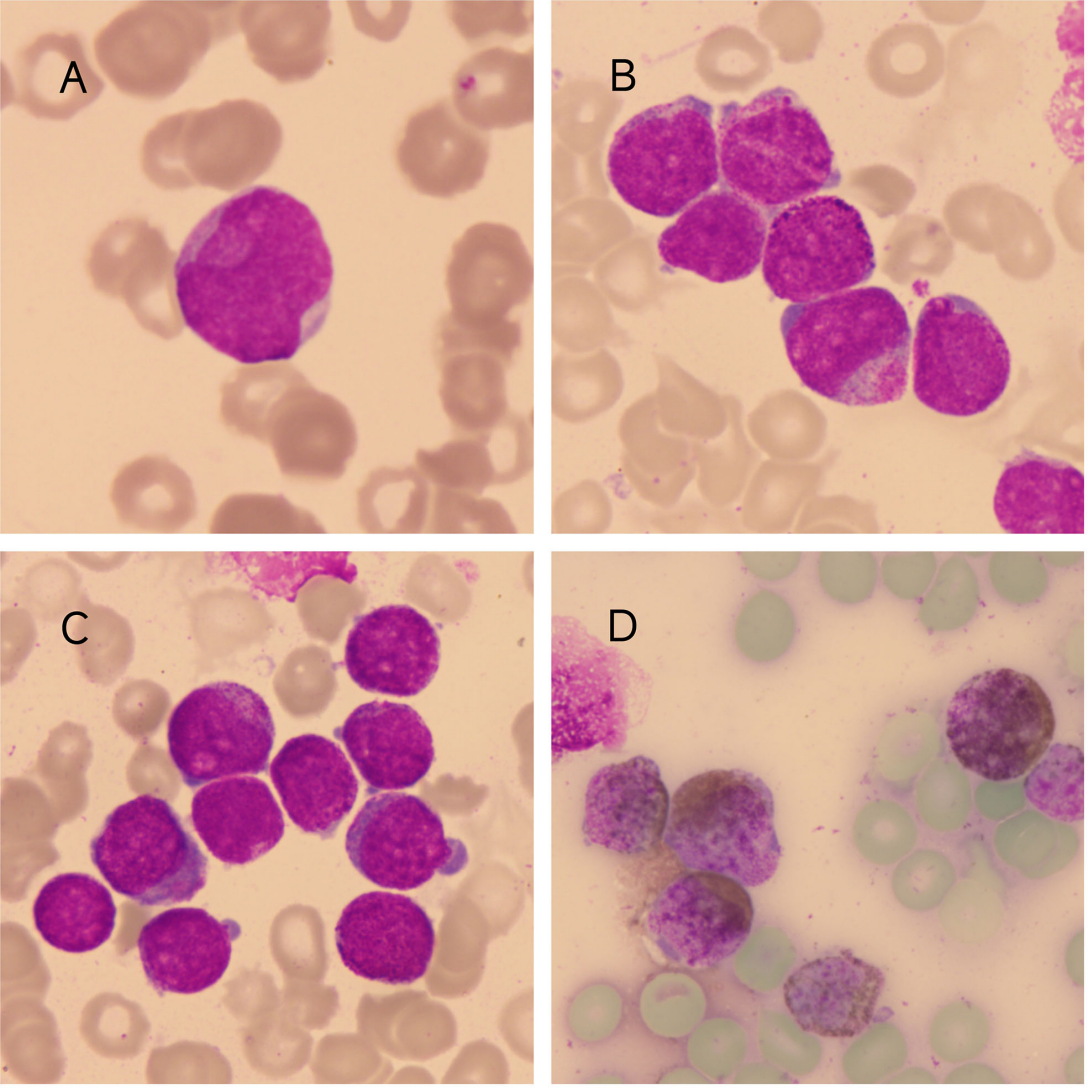
**(A)** Demonstrates cup-like nuclear invaginations in blasts (Wright-Giemsa staining, ×1000). **(B, C)** Bone marrow smear showing blasts with cytoplasmic biphasic differentiation (endoplasm/ectoplasm) and pathognomonic azurophilic granules. (Wright-Giemsa staining, ×1000). **(D)** Myeloperoxidase staining of the blasts was positive (×1000).

A 2-year-and-9-month-old male patient presented with persistent diarrhea and abnormal peripheral blood parameters. Initial laboratory findings included a white blood cell count of 16.94 × 10^9^/L, an absolute neutrophil count of 3.13 × 10^9^/L, hemoglobin of 95 g/L, platelets of 146 × 10^9^/L, and markedly elevated high-sensitivity C-reactive protein at 161 mg/L. Peripheral blood smear analysis revealed 40% blasts, while bone marrow aspiration demonstrated aggressive disease progression with 90% blast infiltration. Morphologically, blasts exhibited distinctive features such as cup-like nuclear invaginations (**Figure 1A**) and abundant azurophilic granules accompanied by heterogeneous cytoplasmic zoning (“inner/outer zones”) (**Figures 1B, C**), mimicking abnormal promyelocytes. Strong myeloperoxidase (MPO) positivity (**Figure 1D**) further supported myeloid lineage differentiation.

**Figure 2:**
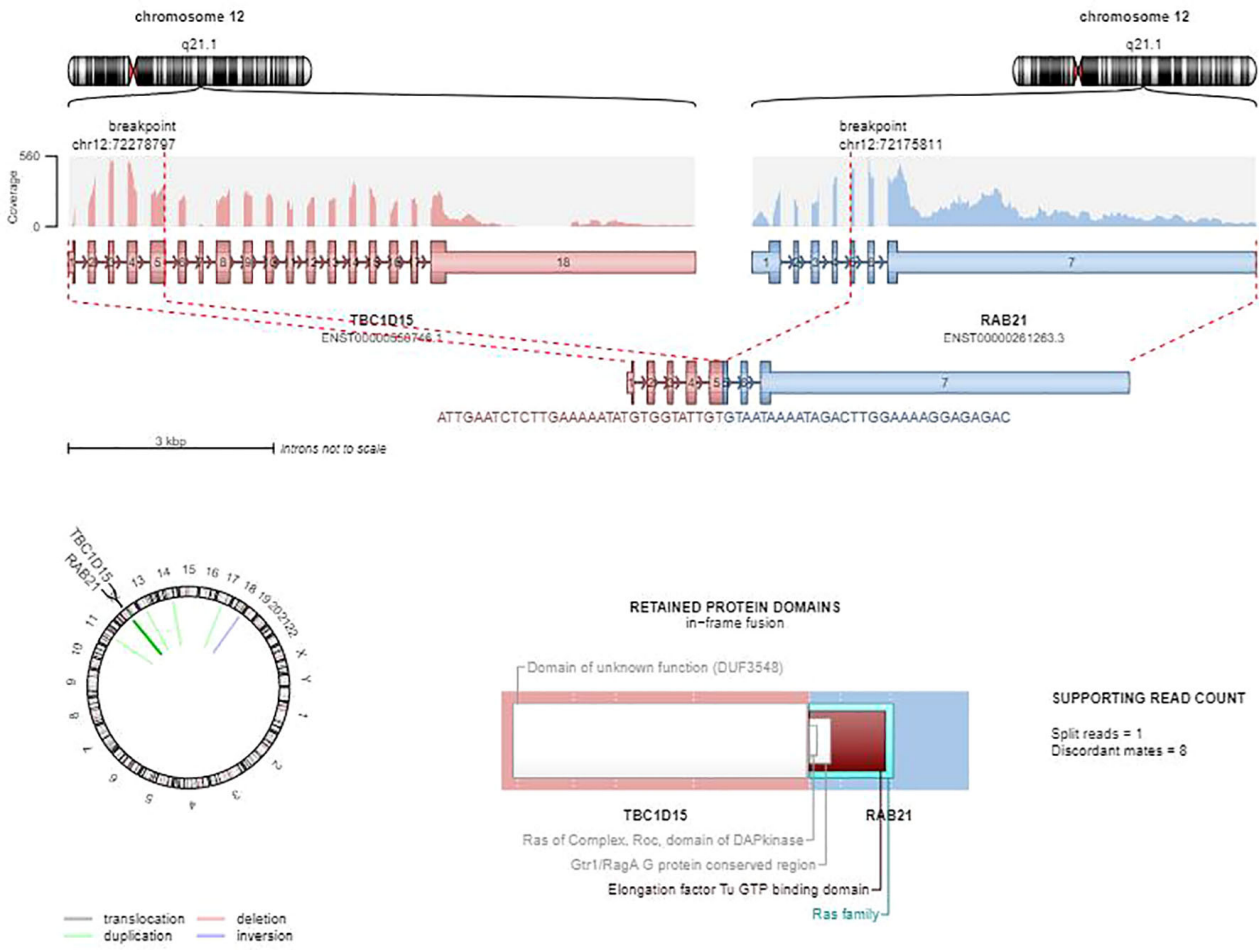
*TBC1D15::RAB21* gene fusion diagram.

Immunophenotypic analysis demonstrated strong CD33 expression, cytoplasmic myeloperoxidase (cMPO) positivity, partial CD45 expression, and weak CD15 expression, while testing negative for CD34, HLA-DR, CD7, CD123, CD38, CD117, CD66c, CD64, CD14, CD11b, CD56, CD19, CD10, sIgM, CD20, CD36, CD58, TdT, CD2, sCD3, cCD3, cCD22, CD9, cIgM, cCD79a, CD4, CD8, CD5, CD16, and CD71. Cytogenetic analysis demonstrated a normal karyotype (46,XY). Molecular profiling was negative for *PML*::*RARA*, *RUNX1*::*RUNX1T1*, and *CBFB*::*MYH11* fusions. However, next-generation sequencing identified a TBC1D15::RAB21 fusion (**Figure 2**), along with pathogenic mutations in FLT3-ITD and *NPM1*. Additionally, two somatic variants of uncertain significance were detected at low allele frequencies: *GATA2* (10% VAF) and *PTEN* (0.4% VAF), whose clinical significance in this context is unclear.

The patient was diagnosed with acute myeloid leukemia (non-APL subtype). Despite morphological overlap with APL—including hypergranular blasts with cup-like nuclei—the absence of *PML::RARA* fusion, combined with immunophenotypic markers (CD33+/cMPO+/CD34−/CD45dim) and molecular findings, supports classification as a rare fusion-driven AML subtype. Concurrent FLT3-ITD (high-risk marker) and *NPM1* mutations indicate a complex prognostic profile, though *NPM1* may partially counterbalance adverse outcomes.

Treatment comprised high-intensity AML chemotherapy (anthracycline/cytarabine-based regimen), achieving initial remission. Subsequent allogeneic hematopoietic stem cell transplantation (allo-HSCT) was performed. At the 6-month post-transplant follow-up, the patient remained in sustained complete remission without evidence of relapse.

## 3 Discussion

The pathogenesis of pediatric AML is predominantly driven by fusion gene events, with critical genetic abnormalities detected in over 90% of cases (2, 15). Major disease subtypes are defined by recurrent fusion genes such as *RUNX1::RUNX1T1* (10%–15%) and *CBFB::MYH11* (5%–10%), which disrupt transcriptional regulation to impair myeloid differentiation (15, 16), while *KMT2A* rearrangements (20%–25%) promote leukemogenesis through epigenetic dysregulation and HOXA cluster activation (1, 14, 17),. These fusion oncoproteins systemically disrupt cell differentiation and proliferation pathways, synergizing with mutations like FLT3-ITD to establish clonal dominance (1, 16, 18). Such molecular aberrations serve not only as diagnostic biomarkers but also as pivotal determinants for prognostic stratification and targeted therapy selection (19).

The rare *TBC1D15::RAB21* fusion identified in this case, it has been previously reported in AML cases that lacked canonical RARA rearrangements but exhibited APL-like features, underscoring its association with this specific morphologic mimicry (12–14). Clinically, its co-occurrence with FLT3-ITD and *NPM1* mutations aligns with the established pattern of rare fusions (e.g., *NUP98::NSD1*) coexisting with kinase-activating mutations, suggesting a potential role as a secondary driver in enhancing leukemic stem cell survival (20, 21). Diagnostically, non-canonical fusions like *TBC1D15::RAB21* pose challenges due to overlapping morphological features (e.g. cup-like nuclear morphology mimicking APL), necessitating comprehensive molecular profiling to resolve diagnostic ambiguities. This emphasizes the critical importance of genomic assays in distinguishing entities like *NUP98::NSD1*-associated AML (often misclassified as AML-M5), thereby guiding precise therapeutic interventions (20).

The FLT3-ITD mutation occurs in 10%–30% of pediatric AML cases and is strongly associated with high relapse risk and poor survival outcomes (6–11). Its oncogenic mechanism involves constitutive kinase activation, leading to uncontrolled cell proliferation through downstream signaling pathways (e.g.STAT5, MAPK) (22, 23). *NPM1* mutations frequently co-occur with FLT3-ITD, where they demonstrate independent prognostic benefits in FLT3-ITD-positive patients by partially mitigating adverse effects (5). Notably, contemporary data suggest that pediatric cases harboring both FLT3-ITD and *NPM1* mutations exhibit favorable clinical trajectories, potentially obviating the need for routine allo-HSCT in selected cohorts (5). In this case, the dual positivity for FLT3-ITD and *NPM1* aligns with this prognostic model, while supporting the successful application of allo-HSCT as consolidation therapy to prevent relapse (24).

In this case, bone marrow blasts displaying cup-like nuclear invaginations and azurophilic granules morphologically mimicked abnormal promyelocytes, a hallmark typically linked to APL with *PML::RARA* fusion (25). While such features strongly suggest APL, FLT3-ITD mutations can coexist with APL-like phenotypes and induce atypical presentations in genuine APL cases (26). Critical diagnostic vigilance is required, as standard APL therapies (arsenic trioxide/retinoic acid) prove ineffective for non-APL AML, whereas FLT3-ITD+ AML necessitates FLT3 inhibitor-based combinations. Although the immunophenotype (CD34−HLA-DR−) partially overlapped with APL, discordant CD117 negativity deviated from classic APL profiles (typically CD117+ in hypergranular subtypes), highlighting the critical need for genetic confirmation (*PML::RARA* testing via FISH/RT-PCR) to prevent treatment delays (27, 28). This diagnostic challenge underscores the necessity of integrating morphological analysis, multiparametric flow cytometry, and molecular diagnostics to resolve mimicry syndromes and guide risk-adapted therapeutic strategies.

FLT3-ITD-positive AML demonstrates suboptimal responsiveness to conventional chemotherapy, necessitating therapeutic augmentation through FLT3 inhibitors (e.g., sorafenib) to overcome tyrosine kinase-mediated resistance mechanisms (26, 29). In the presented case, the observed favorable remission profile may be attributed to the synergistic combination of post-remission allo-HSCT with the prognosis-modulating *NPM1* mutation, which potentially counterbalances FLT3-ITD-driven adverse effects (26). The survival benefit of allo-HSCT in this context likely stems from enhanced graft-versus-leukemia effects targeting residual disease. Future therapeutic strategies should prioritize precision oncology approaches, including RNA sequencing for detecting cryptic fusion transcripts and mandatory molecular verification in cases exhibiting morphologic mimicry, to mitigate diagnostic pitfalls and optimize treatment allocation (30).

## 4 Conclusion

This case of pediatric AML driven by the rare *TBC1D15*::*RAB21* fusion highlights a critical diagnostic pitfall where blast morphology strikingly mimics APL. The potential for misclassification was high and could only be averted through an integrated diagnostic approach. The discordance between the APL-like morphology, the atypical immunophenotype (CD34^-^/HLA-DR^-^/CD117^-^), and the molecular profile (negative for *PML*::*RARA* but positive for *TBC1D15*::*RAB21*, FLT3-ITD, and *NPM1*) underscores the necessity of comprehensive genetic testing in all cases of suspected APL. This case illustrates that morphology alone is insufficient for accurate AML subtyping. Future studies are needed to elucidate the leukemogenic mechanisms of the *TBC1D15*::*RAB21* fusion and its interaction with co-mutations. For now, this report serves as a crucial reminder to maintain a high index of suspicion for morphological mimics and to prioritize rapid, integrated diagnostics to ensure precise, risk-adapted therapy for pediatric AML patients.

## Data Availability

The datasets presented in this study can be found in online repositories. The names of the repository/repositories and accession number(s) can be found in the article/supplementary material.
